# Nitrates Removal from Simulated Groundwater Using Nano Zerovalent Iron Supported by Polystyrenic Gel

**DOI:** 10.3390/polym15010061

**Published:** 2022-12-23

**Authors:** Oanamari Daniela Orbuleţ, Annette Madelene Dăncilă, Simona Căprărescu, Cristina Modrogan, Violeta Purcar

**Affiliations:** 1 Analytical Chemistry and Environmental Engineering Department, Faculty of Chemical Engineering and Biotechnologies, University Politehnica of Bucharest, Gheorghe Polizu Street, No. 1-7, 011061 Bucharest, Romania; 2 Inorganic Chemistry, Physical Chemistry and Electrochemistry Department, Faculty of Chemical Engineering and Biotechnologies, University Politehnica of Bucharest, Gheorghe Polizu Street, No. 1-7, 011061 Bucharest, Romania; 3 National Institute for Research & Development in Chemistry and Petrochemistry—ICECHIM, Splaiul Independentei No. 202, 060021 Bucharest, Romania

**Keywords:** nano zerovalent iron, Purolite A400, nitrate, groundwater, adsorption studies, adsorption mechanism

## Abstract

The main objectives of this present paper were to indicate the immobilization of nano zerovalent iron (nZVI) onto a polymeric material (Purolite A400) and the synthesis of the polymeric material (A400-nZVI) through sodium borohydride (NaBH_4_) reduction. The obtained polymeric material (A400-nZVI) was used for the nitrate ions removal from a simulated groundwater at different conditions. The polymeric materials, without and with nano zerovalent iron (A400 and A400-nZVI), were characterized trough the FTIR, SEM-EDAX, XRD, and TGA analysis. The analysis confirmed the presence of nano zerovalent iron (nZVI) onto the polymeric material (A400). The adsorption capacity of A400-nZVI, used as polymeric adsorbent, was evaluated by kinetic and thermodynamic studies. The obtained experimental results indicated that the nitrate ions reduction was fitted well by models: pseudo-second-order kinetic and Freundlich isotherm. According to the kinetic model results, a reaction mechanism could exist in the stage of reactions. The higher value of removal nitrate (>80%) was obtained under acidic condition. The results indicated that the obtained polymeric material (A400-nZVI) can be considered as a potential polymeric adsorbent for different pollutants from groundwater and wastewater.

## 1. Introduction

Nitrate–nitrogen contamination of global water resources has become an environmental and public health problem worldwide. Although nitrates and nitrogenous compounds are used as important elements in the life process, nitrate is a potential hazard when it is found in drinking water at high enough concentrations [1]. Nitrates act as a precursor to several health hazards: methemoglobinemia (e.g., blue baby syndrome), carcinoma, malformation, and mutation defects [2]. Different biological or chemical techniques were used for the destruction of nitrate in water, such as biological denitrification [3], catalytic denitrification [4], reverse osmosis [5], adsorption [6,7], chemical reduction [8], ion exchange [9], and electrolysis [10]. Among various wastewater treatment methods, chemical reduction is the most favorable method, considering its high effectiveness and permanency. In the literature it was reported that different adsorbent materials (e.g., ion-exchange resin, plants, biochar) have been used for the removal of various pollutants (e.g., heavy metals, dyes) from different wastewaters or groundwaters [6,11,12]. Zaidi et al. [11] demonstrated that the Tarap absorbent can be used for the elimination of dye from simulated wastewater. Kooh et al. [12] showed the adsorption capacity of methylene blue dye utilizing an aquatic plant by machine learning algorithms.

Nanoscale zero-valent iron (nZVI) has been widely investigated for the reduction of numerous contaminants, both organic and inorganic, due to its inexpensiveness, good availability, and stability [13,14,15]. It was reported that the nZVI reactivity can be influenced by some parameters, such as the nature of the iron material, production process, morphologies, crystals nature, and impurities presence [16]. Ratnayake et al. [17] synthesized a stabile nZVI using an inexpensive methodology for removing nitrate from drinking water systems. Wen et al. [18] showed that the nZVI can be utilized as a suitable and useful material for phosphate elimination from contaminated water. Shi et al. [19] reported the immobilized of nZVI in a chelating resin by diminution of NaBH_4_. The results showed that the elimination performance of NO_3_^−^ and Pb^2+^ was 87%, and 97%, respectively. Guo et al. [20] indicated that the system of ZVI/oxidants can be used for removal of nitrogen species at non-acidic pH. Chanthapon et al. [21] synthesized a durable gel-type cation exchange resin that contains nZVI for selective trace Pb^2+^ elimination from the contaminated water. The obtained results indicated that Pb^2+^ adsorption capacity was unaffected by the presence of high concentrations of competing ions (e.g., Na^+^ and Ca^2+^). Wang et al. [22] demonstrated that the pine derived biochar can be utilized as a material to stabilize nZVI for As(V) removal. Yang et al. [23] prepared a ZVI-based agent by pretreating ZVI with H_2_O_2_/HCl and testing its effectiveness by exposure to argon, as well as air. They showed that the extremely efficient nitrate reduction can be achieved over a wide pH range (4–10). The utilization of nZVI inside an ion exchanger (anion or cation resin Purolite) represents an ideal alternative for removal of different pollutants (e.g., metallic ions and dyes) from wastewater or groundwater due to higher reactivity of nZVI and higher selectivity and adsorption efficiency of ion-exchange resin containing specific functional groups (e.g., quaternary ammonium, sulfonic acid, and iminodiacetic acid [13,14,21,24]). Padungthon et al. [24] synthesized a new arsenic adsorbent utilizing a polymeric support that was loaded with nanoparticles of zirconium oxide. They demonstrated that the adsorbent was efficient over numerous cycles of exhaustion–regeneration when the anions were present at high concentrations. Fu et al. [25] realized a resin containing nanoscale zero-valent iron by the method of borohydride reduction. The obtained results reveal that the obtained resin can be used as an efficient agent for treating wastewater that contains Cr(VI) and Cr(III). Ali et al. [26] showed that the iron nanoparticles obtained through the method of ferrous sulfate, incorporated on a porous cation-exchange resin, could be utilized for Cr(VI) reduction. Balan et al. [27] showed that base anionic resins having the structure of gel (Purolite A400 and Purolite A850) can be used for sorption of Cr(VI) from aqueous solutions. Amphlett et al. [28] prepared the synthetic resins and proved that these are effective in the removal of uranyl from aqueous sulfate media.

In the present study, the prepared nano zerovalent iron (nZVI) supported on polymeric material (Purolite A400) was used for removal of nitrate ions from a simulated groundwater. Also, the interaction between nZVI and polymeric material, as well as their removal mechanism, were indicated. The polymeric materials, without and with nZVI, were characterized by Fourier transform infrared spectroscopy (FTIR), scanning electron microscopy (SEM), EDAX spectrometer, thermogravimetric analysis (TGA), and X-ray diffraction (XRD). In addition, the adsorption efficiency of polymeric material with nano zerovalent iron (A400-nZVI) was evaluated.

## 2. Materials and Methods

### 2.1. Materials

Purolite A400 (strongly base anion resin with gel polystyrene crosslinked with divinylbenzene structure, containing quaternary ammonium functional groups, Cl^-^ form) was purchased from Purolite Ltd. (Purolite S.R.L., Bucharest, Romania, an affiliate of Purolite Corporation, King of Prussia, AR, USA). Ferrous sulfate hexahydrate (Fe(SO_4_)·6H_2_O) and sodium borohydride (NaBH_4_) were acquired from Chimopar (Chimopar TRADING SRL, Bucharest, Romania). Ethanol (C_2_H_5_OH) and sulfuric acid (H_2_SO_4_) were purchased from Sigma Aldrich (Merck KGaA, Darmstadt, Germany). All the materials were analytical grade and were used as procured. Distilled water was necessary for obtaining aqueous solutions.

### 2.2. Synthesis of the Polymeric Material

The polystyrenic gel (Purolite A400) containing zerovalent iron nanoparticles (nZVI) was prepared as follows: 45 g of Purolite A400 was added to 250 mL of Fe(SO_4_)·6H_2_O (0.2 M). After 3 h rotation in an end-over-end shaker, the obtained solution was filtered to separate the solid granules, at room temperature (24 ± 1.0 °C), and under nitrogen atmosphere using a nitrogen gas bottle (BOC Linde Company, Distribution Linde Romania, Bucharest, Romania). The obtained solid granules were dried in the oven under vacuum conditions (MultiLab ML-LE 15/11, Distribution Laboratory and Analytical Equipment MultiLab Romania, Bucharest, Romania; IKA-Werke, Inc., Laboratory Equipment, Deutscland, Germany) at 30 °C for 6 h. After this stage, a quantity of 250 mL of NaBH_4_ solution, with a concentration of 0.4 M, was prepared and introduced into a burette to which a round flask with three necks was connected, in which the dry granules were already added. The mixture was stirred using an end-over-end shaker for 3 h under a N_2_ atmosphere. The obtained mixture was kept at room temperature (24 ± 1.0 °C) for 3 h. The borohydride reduction of the ferrous ions is described by the following reaction (1) [29,30,31]:

(1) $$Fe^{3+}+3BH_{4}^{-}+9H_{2}O\to 4Fe^{0}+3H_{2}BO_{3}^{-}+12H^{+}+6H_{2}$$

The zero nanovalent iron supported on polystyrenic gel (A400-nZVI) was obtained through filtration and washed three times using deoxygenated water and then stored in a round-bottomed flask over where the ethanol solution was added (absolute for analysis) to prevent any further oxidation.

**Figure 1:**
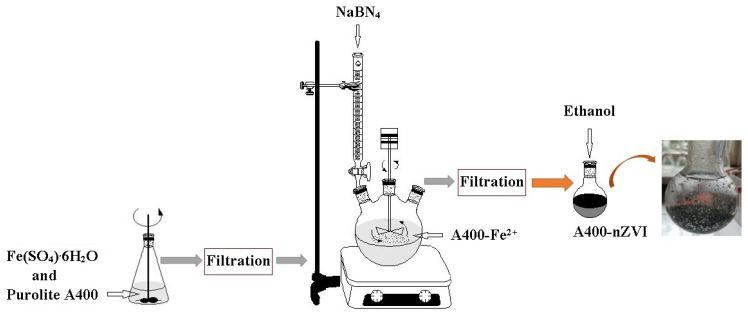
Schematic illustration of synthesis steps to achieve A400-nZVI.

The schematic preparation of A400-nZVI was illustrated in Figure 1.

The polymeric materials, dried under vacuum condition, without and with zerovalent iron nanoparticles (A400 and A400-nZVI), were characterized using different techniques, such as: FTIR, SEM-EDAX, TGA, and XRD analysis. Also, the dried polymeric material A400-nZVI was used as polymeric adsorbent for removal of nitrate ions from a simulated groundwater in order to demonstrate the adsorption capacity. 

### 2.3. Characterization of the Polymeric Materials

Surface morphology of the polymeric materials was performed through a scanning electron microscope (SEM) (Quanta 650 FEG, FEI Company, Hillsboro, OR, USA) equipped with an EDAX spectrometer (175.6 eV resolution) (Oxford Instruments, Hillsboro, AZ, USA).

The infrared spectra of the polymeric materials were registered on a FTIR spectrophotometer (PerkinELMER, Ltd., London, UK) in the transmittance mode equipped with a Golden Gate unite. Data were performed in the wavenumber range between 4000 and 650 cm^−1^, with a resolution of 4 cm^−1^. 

The phase composition analysis of polymeric materials was investigated using an X-ray diffractometer (XRD) equipped with a copper target (X’Pert PRO, PANalytical Co., Almelo, The Netherlands). The XRD experimental data were collected in the 2θ range between 10 and 90 degrees and counting time of 2 s per step. 

TGA analysis of the polymeric materials was performed by a TGA instrument (TA Instruments Q500 TGA, New Castle, AR, USA) equipped with a 16-chamber auto-sampling platform. The samples were heated from 25 to 675 °C, at a heating rate of 10 °C min^−1^ in an inert nitrogen environment. 

### 2.4. Kinetics Studies

The kinetic study experiments were conducted by using simulated groundwater to observe the effect of the A400-nZVI on the nitrate ion removal capacity over time.

Simulated groundwater containing nitrate ions was obtained by dissolving 1.9175 g KNO_3_ into 1000 mL of distilled water (stock solution). The working solutions containing nitrate ions were prepared by diluting 30 mg of A400-nZVI in different amount of stock solution utilizing deionized water and sulfuric acid solution (2 M) to obtain solutions with concentration of 10, 40, and 100 mg L^−1^. The sulfuric acid solution was necessary to adjust the value of pH for the working solutions at 3, using a pH-meter JKPH009 (Shanghai Ltd., Shanghai, China). The obtained solutions in the beakers were stirred at 150 rpm utilizing a magnetic stirrer and placed into an incubator, where the value of temperature (25 °C) was maintained constant. At predetermined times (2, 5, 10, 15, 20, 30, 40, and 60 min), 2 mL of solution were vacuum filtrated using a filter paper (0.22 μm pore size) (Merck SRL, Bucharest, Romania, affiliation with Merck KGaA, Darmstadt, Germany). Afterwards, all solutions were analyzed spectrophotometrically to determine the concentrations of nitrate, nitrites, and ammonium using a UV-VIS spectrophotometer (UV-1900 Shimadzu, Shimadzu Europa GmbH, Duisburg, Germany). The determination of the concentration was carried out at wavelengths of 220 nm for nitrate (according to the standard ASTM 4500 method), 540 nm for nitrite, and 655 nm for ammonium.

For the modeling of the kinetic data, the pseudo-first-order kinetic and pseudo-second-order kinetic models were used, as described by the relations (2) and (3) [31,32,33]:

(2) $$\ln(a_{eq}-a_{t})=\ln(a_{eq})-k\prime \cdot \;t$$

where:

*a_t_* = adsorption capacity of polymeric material A400-nZVI at time t, mg g^−1^;

*a_eq_* = adsorption capacity of polymeric material A400-nZVI at equilibrium, mg g^−1^;

*k^’^* = the adsorption rate constant of pseudo-first-order model, min^−1^;

*t* = stirring time, min.

The pseudo-second-order kinetic model was expressed as (3):

(3) $$\frac{1}{a_{eq}-a_{t}}-\frac{1}{a_{eq}}=k^{\prime \prime }\cdot t$$

where:

*k″* = the rate constant of pseudo-second-order adsorption model, g mg^−1^ min^−1^; 

*a_eq_* = the amount of nitrate ions adsorbed on A400-nZVI at equilibrium, mg g^−1^;

*a_t_* = the amount of nitrate ions adsorbed on A400-nZVI at time t, mg g^−1^.

The speed of the adsorption process (ν) can be calculated using Equation (4):

(4) $$v=\frac{\partial a_{t}}{\partial t}$$

To verify the adsorption efficiency of the synthesized polymeric material, A400-nZVI was utilized as polymeric adsorbent material for nitrate ions removal from a simulated groundwater. The removal adsorption efficiency of the polymeric material A400-nZVI was realized according to a series of physico-chemical parameters: the initial concentration of nitrates in the solution, the initial pH of the aqueous solution, and the reduction speed, respectively. 

The removal efficiency (*η*) of the nitrate ions from the samples is defined as a concentration ratio (Equation (5)) [19,32,33]:

(5) $$\eta \;\left(\%\right)=\frac{C_{i}-C_{f}}{C_{i}}\cdot 100$$

where:

*C_i_* = the initial concentration of nitrate ions from the initial sample, mg L^−1^;

*C_f_* = the concentration of nitrate ions removed from the simulated groundwater, mg L^−1^.

### 2.5. Thermodynamic Studies

The thermodynamic study was realized using a simulated groundwater containing nitrate ions. The experiments were carried out by following procedure: 30 mg of A400-nZVI was added in a glass flask and dissolved in 30 mL of solution containing nitrate ions (stock solution). The stock solution was diluted to obtain solutions with concentrations of 10, 20, 40, 60, 80 and 100 mg L^−1^, at pH = 3. The solutions were stirred in a shaker at a speed rate of 150 rpm for 4 h until equilibrium was reached. The flasks were placed in an incubator where the temperature was kept constant at 25 °C. 

The adsorption capacity of A400-nZVI at equilibrium, at different concentrations, were described by Langmuir and Freundlich adsorption isotherms.

The Langmuir adsorption isotherm model (6) is expressed as (6) [19,20,32]:

(6) $$a=\frac{K_{L}\cdot C_{e}\cdot a_{eq}}{1+K_{L}\cdot C_{e}}$$

where:

*a* = adsorption capacity of polymeric material A400-nZVI at equilibrium, mg g^−1^;

*K_L_* = the Langmuir constant related to the adsorption capacity, L mg^−1^;

*C_e_* = the equilibrium concentration of nitrate ions in the aqueous phase, mg L^−1^;

*a_eq_* = the adsorption capacity at equilibrium of A400-nZVI, mg g^−1^.

The Freundlich adsorption isotherm model (7) is expressed as (7) [19,20,21,32]:

(7) $$\ln a_{eq}=\ln K_{F}+\frac{1}{n}\ln C_{e}$$

where:

*C_e_* = the concentration at equilibrium of the nitrate ions in the aqueous phase, mg L^−1^;

*a_eq_* = the adsorption capacity at equilibrium of A400-nZVI, mg g^−1^; 

*K_F_* = the Freundlich constant related to the adsorption capacity, (mg g^−1^)/(mg L^−1^)^n^;

*n* = an empirical parameter;

*1/n* = m is a constant indicating the Freundlich isotherm intensity.

The adsorption capacity at equilibrium of A400-nZVI is expressed by Equation (8) [21]:

(8) $$a_{eq}=\frac{\left(C_{0}-C_{e}\right)}{m}V$$

where:

*C_0_* = the initial concentration of solution at initial time, mg L^−1^;

*C_e_* = the concentration of solution at equilibrium, mg L^−1^;

*m* = the amount of polymeric material A400-nZVI, mg;

*V* = the volume of solution, mL.

## 3. Results and Discussions

### 3.1. Characterization of Polymeric Materials

#### 3.1.1. SEM-EDAX

**Figure 2:**
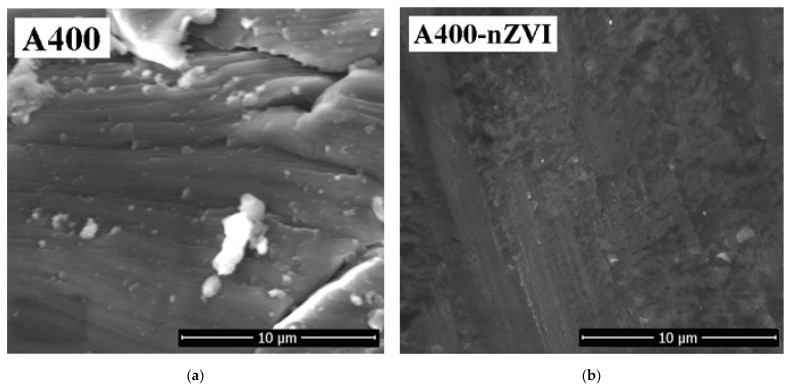
SEM images (at a magnification of 5000×) of polymeric materials: (**a**) A400 and (**b**) A400-nZVI.

**Figure 3:**
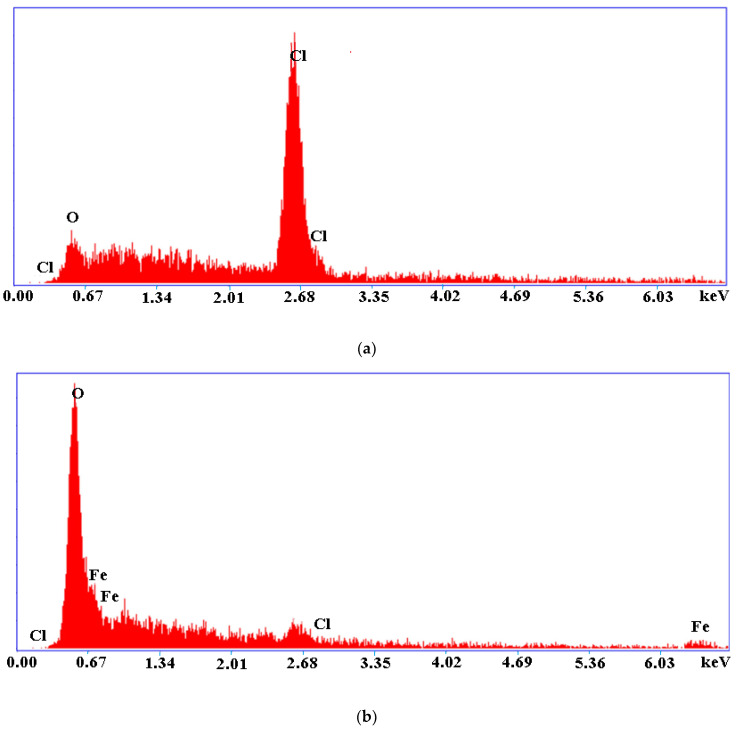
EDAX spectra of polymeric materials: (**a**) A400 and (**b**) A400-nZVI.

The SEM images (magnification of 5000×) and EDAX spectra of polymeric materials, A400 and A400-nZVI, are indicated in Figure 2 and Figure 3.

The polymeric materials surface was realized through SEM to verify the dispersion and the fixation of iron ions at the polymeric material A400-nZVI surface. The SEM images suggest that the polymeric materials have a porous structure (Figure 2). The A400 surface (Figure 2a) is very irregular. The SEM image of the A400-nZVI (Figure 2b) shows that the surface is dense and compact, and after this, the nZVI particles were fixed in the A400 polymeric matrix. Also, images evince that the nZVI dispersed on the A400 surface are presented in several nanometer sizes, with diameter between approximately 75 and 150 nm. The difference between the size of the nZVI particles can be assigned to the concentration of the sodium borohydride solution [18,20]. SEM results showed homogeneous lusters of iron oxidation products at the surface of A400-nZVI. 

Jiang et al. [13] related that the concentration of NaBH_4_ influence the homogeneity of Fe distribution in the obtaining the nZVI–resin hybrids. They observed that more iron ions were distributed in the vicinity of the nucleus region of the obtained hybrid at a high solution concentration of NaBH_4_ (mass concentrations of 7.2%).

EDAX analysis was realized to evince the interaction of nZVI with polymeric material A400.

EDAX illustrated that sample A400 was composed of Cl, and O. The calculated quantitative percentages were Cl 80.26%, and O 19.74%. The EDAX for A400-nZVI indicate the presence of Fe, O, and Cl elements. The calculated quantitative percentages were Fe 31.81%, O 55.40%, and Cl 12.80%. It can be observed that the quantitative percentage is higher, which demonstrate the presence of nZVI into the polymeric material. The presence of peaks for Fe in the polymeric material A400-nZVI can be attributed to the oxidized iron. Also, the presence of peaks for Fe demonstrated that the polymeric material A400-nZVI surface consisted of an oxide film layer that can occur throughout the dried process in vacuum conditions [19]. These observations were consistent with the results obtained from SEM and XRD analysis.

#### 3.1.2. FTIR Spectroscopy

**Figure 4:**
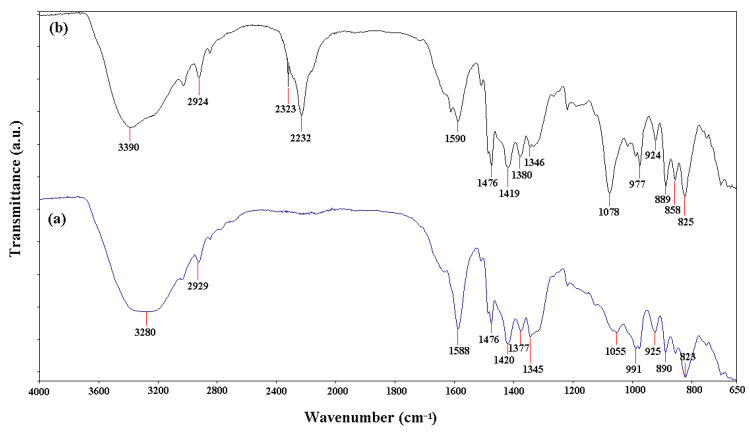
FTIR spectra of polymeric materials: (**a**) A400 and (**b**) A400-nZVI.

The FTIR spectra of the polymeric materials (A400 and A400-nZVI) are shown in Figure 4. The differences between polymeric materials in the structural bonds can be observed. 

In Figure 4, it can be observed that the strong peak at 3280 cm^−1^ (sample A400), attributed to OH groups (bending and stretching vibrations) [19,33], was shifted to higher wavenumber value of 3390 cm^−1^ (sample A400-nZVI), which indicated the presence of interstitial water molecules.

The FTIR spectrum of A400-nZVI showed a peak at 2323 cm^−1^ that was attributed to B–O bending vibration, resulting from the reduction of iron ions following the use of NaBH_4_ solution [19,34]. Also, a detected peak at 2232 cm^−1^ (sample A400-nZVI) corresponds to the stretching vibration of the –C≡N bond (stretching vibration) from the nitrile group [33].

Comparing the FTIR spectra of samples A400 and A400-nZVI, it can be observed that the bands at 1588, 1377, and 1055 cm^−1^ were shifted to higher wavenumber values (1590, 1380, and 1078 cm^−1^, respectively), possibly due to the coordinative bond between A400 (polystyrene crosslinked with divinylbenzene) and iron. The peaks at 1476 and ~1590 cm^−1^ were attributed to aromatic stretching vibrations from the quaternary ammonium (functional groups from A500). The peak at ~1346 cm^−1^ was attributed to symmetric carbonyl group. The peak at 1055 cm^−1^ (sample A400) was shifted to higher wavenumber at 1078 cm^−1^ (sample A400-nZVI) and attributed to C–O vibration. This difference confirmed that the A400-nZVI containing nano zerovalent iron and the polymeric materials were prepared using NaBH_4_ [23]. The appearance of peaks at 2929 cm^−1^ (sample A400), and 2924 cm^−1^ (sample A400-nZVI) may be due to the groups –CH_2_– asymmetric stretching vibration, C–H bonds from A400, as well as polymer chains. The peaks at 858 and 825 cm^−1^ were attributed to Fe-O and Fe-O-Fe stretching vibration of Fe_2_O_3_ and Fe_3_O_4_ [19,30,33]. These results reveal that nZVI had been loaded successfully onto the surface of polymeric material A400. The results obtained are in agreement with the EDAX and XRD analyses.

#### 3.1.3. XRD Diffraction

**Figure 5:**
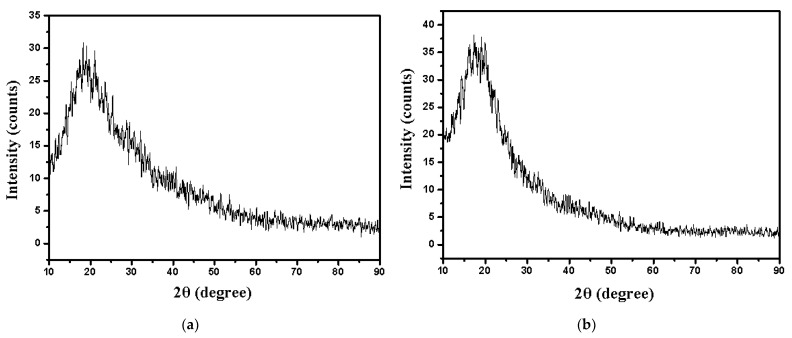
XRD spectra of polymeric materials: (**a**) A400, and (**b**) A400-nZVI.

XRD analysis was realized to elucidate that the nZVI can be incorporated into polymeric material A400 (Figure 5).

The XRD diffraction pattern for the A400-nZVI shows two peaks at 2θ = 44.8° and 82.3°, indicating the presence of nZVI on the A400 surface. The crystal phases of A400-nZVI was confirmed by the presence of nZVI, Fe(OH)_2_ at 2θ = 19.23°, 37.12°, 50.97 °, 56.02°, and 69.58°, and carbon at 2θ = 43.91° and 75.37°. He et al. [26] identified crystal phases for nanoscale zerovalent iron/nickel supported on zeolite at 2θ = 44.8°, and 69.0°. Their results indicate the existence of zerovalent iron on the zeolite surface.

The all position (2θ), d-spacing (d) values, and lattice parameters (a, b, c; α, β, γ) for polymeric material A400-nZVI are indicated in Table 1.

In the synthesis procedure of the A400-nZVI polymer material, the particles were not in contact with atmospheric air. However, there is a possibility to forming an oxide/hydroxide layer around the particles due to the reaction with atmospheric oxygen. The Fe(OH)_2_ phase was observed, which could be due to the exhibition of the particles to the working conditions during the XRD analysis. 

#### 3.1.4. TGA Analysis

**Figure 6:**
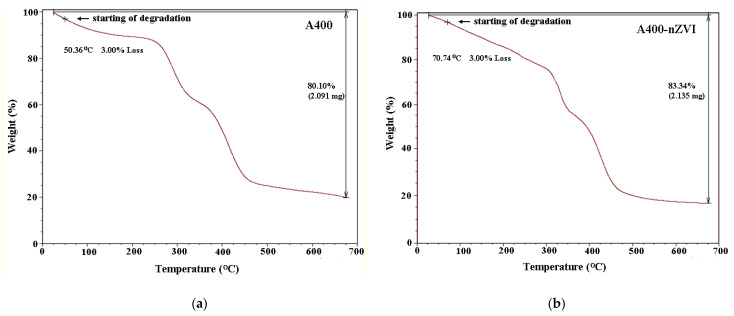
TGA curves of polymeric materials: (**a**) A400, and (**b**) A400-nZVI.

TGA analysis of polymeric materials (A400 and A400-nZVI) was performed from 25 to 650 °C in a nitrogen atmosphere (Figure 6).

The TGA curves of polymeric materials (Figure 6) are different due to the presence of nZVI in the polymeric material A400. It was observed that there are more stages of decomposition. The first stage (25–100 °C) refers to any residual water that is discharged from the resins. Other steps refer to the breakdown/decomposition of the resin itself. Losses of mass in the range (230–480 °C) are evidence of functionalization. As the mass of nZVI-functionalized groups increases, so does the percentage of mass lose from the polymeric resin. In both samples, following thermal decomposition, species containing nitrogen were observed to be released from A400 immediately above 200 °C, and species containing carbon were released at approximately 350 °C. The TGA profiles of the polymeric materials indicate a low percentage weight loss of 3% at low temperature.

From Figure 6, it can be remarked that A400 sample degraded around ~50 °C, while A400-nZVI sample starts to degrade at ~70 °C. The A400-nZVI sample had a residual mass at 700 °C of 83.34%, in comparison with sample A400, that has a residual mass of 80.10%. This result confirmed the association of the iron particles with the surface of A400 resin [30], as well as the presence of residual iron base in the polymeric material A400-nZVI.

### 3.2. Kinetics Evaluation

Kinetics studies indicate information on the adsorption capacity of A400-nZVI for nitrate ions reduction from aqueous solutions. The value of adsorption capacity at equilibrium, a_eq_, and the values of the correlation coefficients are presented in Table 2.

The equilibrium data derived from the conducted batch experiments (under the most favorable conditions) were fitted to both pseudo-first and pseudo-second order kinetic models. According to the correlation coefficients (R^2^), the experimental data were better evaluated by pseudo-second-order kinetic model. The results obtained in this study confirm that the value of the correlation coefficient is high (R^2^ > 0.99), which leads to the idea that the ion exchange process takes place with high intensity, being the main process by which nitrates are eliminated. The nitrate removal percentage was approximately 99% at the low initial nitrate concentration (10 mg L^−1^). Su and Puls [35] studied the effects of different ligands regarding the reduction of nitrate using zerovalent iron. From the correlation analysis, it was found that, for the ligands, there is a negative connection between the reduction rates. The correlation coefficients for the soluble complexes of the ligands with iron ions were R^2^ = 0.701 for Fe^2+^ and R^2^ = 0.918 for Fe^3+^. He et al. [33] reported the kinetic studies using zeolite supported on nano zerovalent iron/nickel. The experimental data indicated that the nitrate remediation and phosphate removal were better fitted by first-order and pseudo-second-order models where the values for correlation coefficients were higher (R^2^ > 0.99).

From the analysis of the kinetic parameters (Table 2), it was observed that the values for k″ are higher at low concentration, followed by the decrease in the values with the increase in the concentration of the solution. The decrease in the k″ value could be due to the limitation of mass transfer. As the adsorption reaction takes place, the nZVI particles that have better pore accessibility will be depleted faster. Later, nZVI adhered in the pores with the limitation of mass transfer, which is indicative of starting to participate in the reaction. The values of k″ vary considerably depending on the concentration of the solution.

It was also highlighted that the nitrate removal increased significantly within 10 min, and then the equilibrium state is reached. These results can be explained, on the one hand, by admitting that the increase in H^+^ concentration favors the redox reaction between nZVI and nitrate ions and prevent the precipitation of ferrous or ferric ions on the surface of A400-nZVI (iron fouling) and, on the other hand, by considering that the polymeric material (A400) has a high affinity for nitrates in a slightly acidic conditions and for relatively low concentrations of these ions in the aqueous solutions. 

Xiaomeng et al. [36] realized the kinetic study on the nitrate and observed that the concentration of nitrate ions was better fitted by pseudo-first-order model. The experimental studies indicated that the reaction rate constant was independent of the initial nitrate concentration. They related that the total denitrification for concentrations of nitrate between 50 and 400 mg L^−1^ occurred after 120 min. 

The obtained results indicate that the rate of nitrate reduction with A400-nZVI is higher for A400-nZVI in the absence of co-ions.

**Figure 7:**
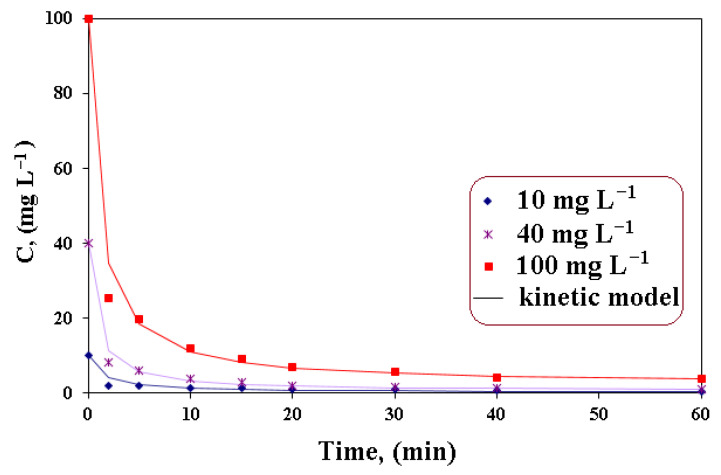
Variation of concentration in time for A400-nZVI.

From Figure 7, it can be observed that the most significant drop in concentration occurs in the first 5–10 min and is very visible at high concentrations of 100 mg L = 1. After ten minutes, the nitrate ion concentration in the solution decreases by 10%, then the decrease is slow, becoming constant after an hour. The downsizing process continued, but at a much slower rate. He at al. [33] studied the removal efficiency of nitrate and phosphate from aqueous solution using nanoscale zerovalent Fe/Ni supported on zeolite. It was related that the maximum values of removal efficiency were 72.5% for nitrate and 98% for phosphate at pH of 3 after 6 h.

The speed rate of nitrate reduction was very fast in the initial period, then the reactivity was lower after a certain time.

**Figure 8:**
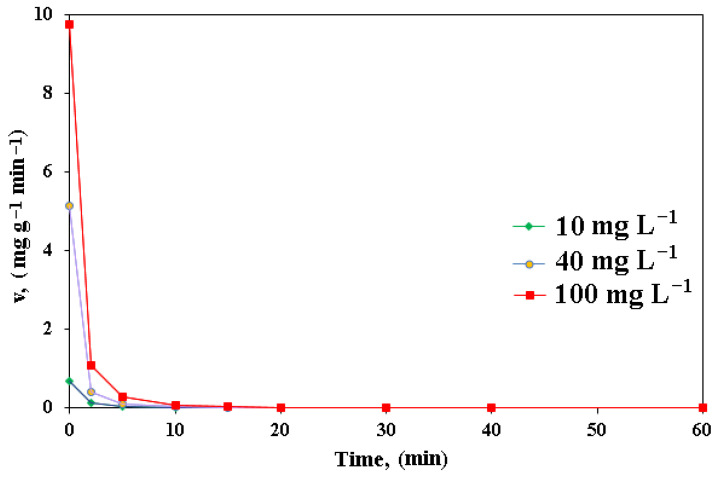
Variation of speed rate in time for A400-nZVI.

As can be seen in Figure 8, nitrate ions were removed by A400-nZVI in a proportion of 70%. It has been reported in the specialized literature that some anions are frequently presented, both in surface waters and in wastewaters, which may interfere with the removal of nitrates by passivating the nZVI surface, competing for surface sites. [37,38]. Reinsch et al. [39] indicated that the nitrate could passivate the nZVI surface for a period of six months, at a higher concentration (5 mM, 70 mgN NO_3_^–^ L^−1^), synthesizing a protective layer of oxide (Fe^3+^) of an insoluble surface formed mainly from maghemite and hematite. He et al. [26] demonstrated the performance of zeolite supported zerovalent Fe/Ni nanoparticles for removal of nitrate at different concentrations of aqueous solution. They reported that the increase of initial nitrate concentration, as well as the removal efficiency of nitrate, decreased due to the restricted number of functioning sites. Also, it was observed that the increase in the initial concentration of nitrate led to rapidly increasing pH in the solution. This fact can be due to the higher nitrate concentration. When zeolite supported zerovalent Fe/Ni nanoparticles react faster with nitrites, the more Fe^0^ and H^+^ are diminished. 

Zhang et al. [40] reported that the nitrate removal efficiency increases with increasing concentrations of solution. The higher value of removal efficiency (94%) was obtained after 120 min. at a solution concentration of 100 mg L^−1^, as well as a concentration of zerovalent iron of 0.416 g L^−1^. Su and Puls [35] demonstrated that the zerovalent iron can be successfully used as a permeable reactive blockade for nitrate removal from groundwater. From the experimental studies, it was observed that small fractions of nitrate were quickly eliminated by zerovalent iron in the first minutes, but the complete nitrate reduction occurs after 120 h due to the passivation of zerovalent iron. 

**Figure 9:**
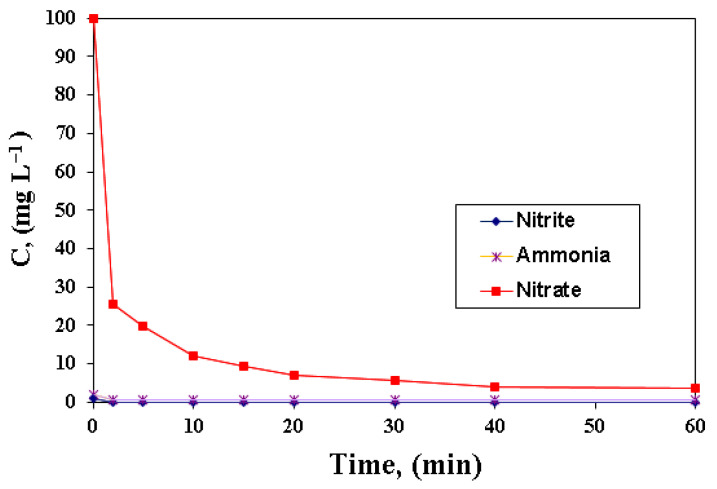
Variation of nitrate, nitrite, and ammonium concentration for removal of NO_3_^−^ on A400-nZVI.

The amount of nitrate, ammonium, and nitrite over time for nitrate reduction on A400-nZVI is shown in Figure 9.

From Figure 9, it can be seen that nitrite ions were not detected in the analyzed solutions. This fact can be explained by the fact that denitrification by nZVI occurred rapidly, and nitrite ions were quickly converted to ammonium ions. It is also noted that the concentration of ammonium ions was not detected, and this can be attributed to the adsorption by the polymeric material A400-nZVI [38,39,40]. The experimental results revealed that A400-nZVI has a high efficiency for the removal of nitrates from aqueous solutions, especially under acidic conditions. In this respect, it was found that over 80% of the nitrate is removed at a pH value of initial nitrate solution of 3, corresponding to an initial concentration of nitrate in the initial solution ranging from 10 to 100 mg L^−1^. Shi et al. [41] studied the ability of zero-valent iron nanoparticles (NZVI) supported on a chelating resin DOW 3N (NZVI-DOW 3N) to remove nitrate from aqueous solution. They related that the NZVI-DOW 3N presented a higher removal efficiency for nitrate (94%) within 480 min. Additionally, it was related that the reduction reaction of nitrate from aqueous solution is complicated due to the possible intermediates and products (e.g., NO_2_^−^, NO, N_2_O, N_2_, N_2_H_4_, and NH_4_^+^) [35]. 

The effect of solution pH on nitrate reduction by A400-nZVI was indicated in Table 3.

In previous works, it has been reported that an acidic pH of aqueous solutions conducts to a rapid decrease in nitrates and a strong activity of the materials. It was also demonstrated that the reduction of nitrites using nZVI is favorable when the pH of the solution is low, according to reaction mechanism proposed and indicated as [20,35]:

(9) $$NO_{3}^{-}+4Fe^{0}+10H^{+}\to NH_{4}^{+}+4Fe^{2+}+3H_{2}O$$

In the literature, it was reported that the neutral or alkaline pH of aqueous solution is unfavorable for reduction of nitrates [26]. The reaction is stopped when the aqueous solution has a pH > 4 [33,38]. 

The pH values increased during the NO_3_^−^ reduction reaction from 3 to 9.37 with increase in the concentration. The results indicate that the total amount of nitrogen species in solution decreased compared to the initial amount. This decrease can be due to the formation of gaseous ammonia and/or gaseous nitrogen or the adsorption of NO_3_^−^ and NO_2_^−^. The pH values in the solution after the reduction of NO_3_^−^ are always higher than before the process, suggesting that the reduction reaction results in the release of OH- ions in the solution. The faster nitrate reduction at low pH can be attributed to the higher production of H_2_, which restored the reactivity of the nanoparticle surface. Low pH values of the solutions led to increased nitrate removal rates. However, the removal efficiency of nitrates was constant in the case of synthetic solutions containing nitrates. At the solution pH > 9, NH_4_^+^ will be changed into NH_3_, which will make adsorption very difficult on the polymeric material A400-nZVI [20,33].

### 3.3. Thermodynamic Evaluation

The experimental data obtained on the adsorption of nitrate ions on the polymeric material A400-nZVI were fitted by the Langmuir and Freundlich isotherm models. The relevant kinetic parameters, obtained using the Langmuir and Freundlich isotherm models, are indicated in Table 4.

**Figure 10:**
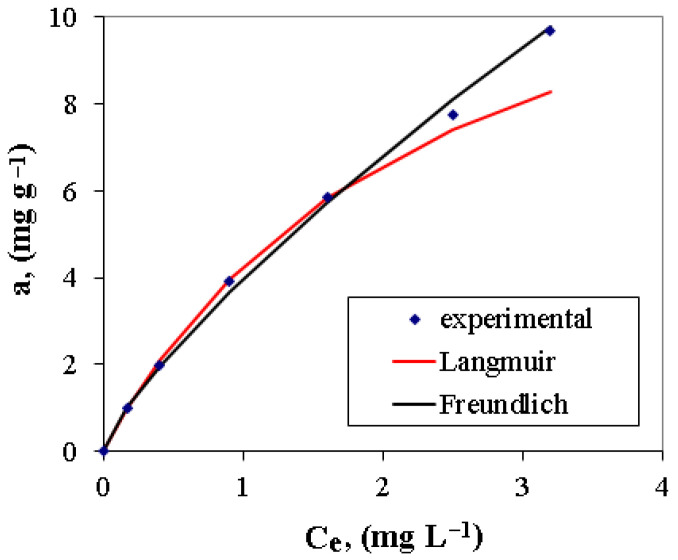
Langmuir and Freundlich isotherms for polymeric material A400-nZVI.

The experimental data of adsorption nitrate ions on the polymeric material A400-nZVI fitted by indicated thermodynamic model, using regression function, are presented in Figure 10. The thermodynamic data indicated that the Freundlich adsorption isotherm model are better fitted in comparison with the Langmuir adsorption isotherm model, suggesting that nitrate ions reduction can be a result of multilayer adsorption occurring on the polymeric material A400-nZVI surface [21]. The best value of the regression correlation coefficient (R^2^ = 0.9974) was obtained for the Freundlich isotherm model. The experimental data and the calculated value of regression correlation coefficient are in good agreement with the calculated values and with the pseudo-second order kinetic model.

### 3.4. Adsorption Mechanism

A frequent mechanism for the removal of nitrates is adsorption [7,32]. In the adsorption process, the nitrate molecule is anchored to the surface by physical or chemical sorption.

**Figure 11:**
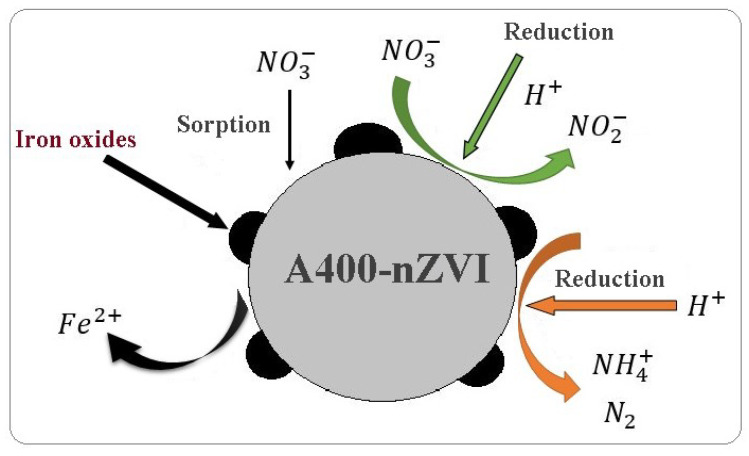
Schematic illustrating: interactions and reactions that take place on the surface of A400-nZVI.

The process by which the A400-nZVI interact with the nitrate ions and the reactions that take place on the A400-nZVI surface are illustrated in the Figure 11.

The oxidation state of the polymeric material A400-nZVI surface depends on the stability and reactivity of the iron. Following the results obtained, the possible stages for the removal of nitrates from groundwater are proposed in reactions (10)–(16) [20,41,42]:

Fe^0^ + 2 H_2_O → Fe^2+^ + H_2_ + 2OH^−^, (10)

(11) $$5\;{\mathrm{Fe}}^{0}\;+2\;{\mathrm{NO}}_{3}^{-}+12\;H^{+}\;\to \;{\mathrm{Fe}}^{2+}+H_{2}\;+\;6H_{2}O$$

(12) $$4\;{\mathrm{Fe}}^{0}+2\;{\mathrm{NO}}_{3}^{-}+10\;H^{+}\;\to \;4\;{\mathrm{Fe}}^{2+}+4\;{\mathrm{NH}}_{4}^{+}+3H_{2}O$$

(13) $${\mathrm{Fe}}^{0}+2\;{\mathrm{NO}}_{3}^{-}+4\;H^{+}\;\to \;{\mathrm{Fe}}^{2+}+2\;{\mathrm{NO}}_{2}^{-}+2H_{2}O$$

(14) $$2{\mathrm{Fe}}^{0}+3{\mathrm{NO}}_{3}^{-}+6H^{+}\to 2{\mathrm{Fe}}^{3+}+3H_{2}O+3{\mathrm{NO}}_{2}^{-}$$

(15) $$2{\mathrm{NO}}_{2}^{-}+8H^{+}+6e^{-}\to N_{2\left(g\right)}+4H_{2}O$$

(16) $$6\;{\mathrm{Fe}}^{2+}+{\mathrm{NO}}_{2}^{-}+8\;H^{+}\;\to \;6\;{\mathrm{Fe}}^{3+}+{\mathrm{NH}}_{4}^{+}+2H_{2}O$$

Iron particles are deposited on the polymeric material A400. The reactions indicated that the Fe^0^ was oxidized at Fe^2+^, at the beginning, and after that, Fe^0^ with NO_3_**^−^** was diminished into NO_2_**^−^** and NH_4_^+^. The reactions that take place for nitrate removal from groundwater using polymeric adsorbent indicated that the nitrate in the solution is adsorbed, and it reacts with the zerovalent iron, resulting in secondary products of the reduction reaction in the aqueous solution: nitrite, ammonium, and gaseous nitrogen. The formation of nitrite and its subsequent removal are indicated in Equations (13)–(15) [20]. The activated hydrogen on the particle sites can absorb the nitrite oxygen, leaving the two consecutively bonded nitrogen atoms that will form nitrogen gas [41]. Interference of redox reactions can occur if the groundwater is oxygenated. Oxygen can oxidize iron causing the formation of solid precipitates of FeO(OH) or Fe(OH)_3_. In addition to the fact that in this case Fe^0^ is no longer available to interact with contaminants, the formation of these solids can block the medium [33,41]. 

## 4. Conclusions

In this work, a polymeric material (A400-nZVI) was successfully synthesized in order to remove nitrate from simulated groundwater.

The FTIR spectra indicated that nZVI was successfully loaded onto polymeric material A400. The SEM image of A400-nZVI showed that nZVI particles, with diameter sizes between 75–100 nm, were fixed in the A400 polymeric matrix, and the A400-nZVI surface is dense and compact, after which the nZVI particles were fixed in A400 polymeric matrix. EDAX indicated the presence of peaks for Fe in A400-nZVI polymeric material. The existence of nano zerovalent iron on A400-nZVI surface was confirmed by XRD.

The kinetic study indicated that the reduction of nitrates was fitted well by pseudo-second-order kinetic model and the higher removal nitrate occurred under acidic conditions (>80%). The experiments indicated that the faster nitrate reduction occurred at low pH due to the higher production of H_2_, which restored the reactivity of the nanoparticle surface. The thermodynamic study showed that the adsorption of nitrate ions on A400-nZVI polymeric material was well fitted by the Freundlich isotherm model.

The structural properties and higher efficiency of A400-nZVI indicated that the synthesized polymeric material could be used for the removal of other pollutants (e.g., dyes and metallic ions) from different groundwaters and wastewaters.

## Figures and Tables

**Table 1:** 2θ, d, and lattice parameters of A400-nZVI.

Phase	System	2θ (°)	d (Å)	Lattice Parameters
C	Cubic	43.91	2.06	a (Å) = 3.5597 b (Å) = 3.5597 c (Å) = 3.5597 α (°) = 90 β (°) = 90 γ (°) = 90
		75.37	1.26	
		92.09	1.07	
		119.88	0.89	
		139.89	0.82	
Fe(OH)_2_	Hexagonal	19.23	4.61	a (Å) = 3.2700 b (Å) = 3.2700 c (Å) = 4.6200 α (°) = 90 β (°) = 90 γ (°) = 120
		31.70	2.82	
		37.12	2.42	
		50.97	1.79	
		56.02	1.64	
		60.02	1.54	
		69.58	1.35	
		79.07	1.21	

**Table 2:** Kinetics parameters for A400-nZVI.

A400-nZVI							
pH	C (mg L^−1^)	Pseudo-First-Order		Pseudo-Second-Order			
		k^’^	R^2^	k″	v (mg g^−1^ min^−1^)	a_eq_ (mg g^−1^)	R^2^
3	10	0.0585	0.7099	0.7201	0.6902	0.9790	0.9988
	40	0.0601	0.7131	0.3300	5.1443	3.9479	0.9999
	100	0.0623	0.7409	0.1019	9.7601	9.7859	0.9999

**Table 3:** Behavior of pH values in solution before and after removal NO_3_^−^ on A400-nZVI.

C, mg L^−1^	pH_initial_	pH_final_
10	3	8.80
40	3	9.05
100	3	9.37

**Table 4:** Thermodynamic parameters for A400-nZVI.

A400-nZVI						
pH	Langmuir			Freundlich		
	K_L_ (mg^−1^ L)	a_eq_ (mg g^−1^)	R^2^	K_F_	m	R^2^
3	0.4260	14.3703	0.9863	3.9819	0.7735	0.9974

## Data Availability

The data presented in this study are available on request from the corresponding author.
